# Advancing Atmospheric Detection of Weakly Absorbing Reactive Trace Gases Using the FY-3E/HIRAS-II TIR Sounder on a Dawn–Dusk Orbit

**DOI:** 10.1021/acs.estlett.5c00501

**Published:** 2025-06-24

**Authors:** Zhenxing Liang, Dasa Gu, Rui Li, Jian Liu, Chengxing Zhai, Hui Su, Alexis K. H. Lau

**Affiliations:** † Division of Environment and Sustainability, 58207 The Hong Kong University of Science and Technology, Clear Water Bay, Hong Kong SAR 999077, China; ‡ Guangdong-Hongkong-Macau Joint Laboratory of Collaborative Innovation for Environmental Quality, 58207 The Hong Kong University of Science and Technology, Clear Water Bay, Hong Kong SAR 999077, China; § School of Earth and Space Science, CMA-USTC Laboratory of Fengyun Remote Sensing, Deep Space Exploration Laboratory, 12652 University of Science and Technology of China, Hefei 230026, China; ∥ College of Environment and Ecology, Shanxi Key Laboratory of Complex Air Pollution Control and Carbon Reduction, 47846 Taiyuan University of Technology, Taiyuan 030024, China; ⊥ Division of Emerging Interdisciplinary Areas, 58207 The Hong Kong University of Science and Technology, Clear Water Bay, Hong Kong SAR 999077, China; # Department of Civil and Environmental Engineering, 58207 The Hong Kong University of Science and Technology, Clear Water Bay, Hong Kong SAR 999077, China

**Keywords:** satellite remote sensing, formic acid, methanol, ammonia, peroxyacetyl nitrate, ethylene, acetylene, isoprene

## Abstract

Weakly absorbing reactive trace gases play important roles in the atmospheric environment and usually have short lifetimes ranging from seconds to days. HIRAS-II, the second hyperspectral infrared atmospheric sounder aboard the world’s first civilian meteorological satellite in dawn–dusk orbit, FengYun-3E (FY-3E), can theoretically detect more than a dozen weakly absorbing reactive trace gases and make important contributions to global trace gas mapping by filling the gap for diurnal variation. This study uses state-of-the-art weak absorber thermal infrared spectral feature quantification and identification methods to detect weak absorbers from FY-3E/HIRAS-II and successfully capture 14 species from 35.4 million FY-3E/HIRAS-II clear-sky measurements in July 2023. We map the reliable global distribution of spectral features from nine routine reactive gases and find that these gases originate from scenes that are usually of special concern, including densely populated areas, vegetation, and biomass burning. This study confirms the capability of FY-3E/HIRAS-II in detecting weak absorbers and serves as a stepping stone for subsequent research in concentration retrieval. The case of the ammonia column over wildfires retrieved using neural network technology initially demonstrates that FY-3E/HIRAS-II can improve our understanding of the diurnal variation of trace gases by complementing measurements at dawn and dusk.

## Graphical abstract

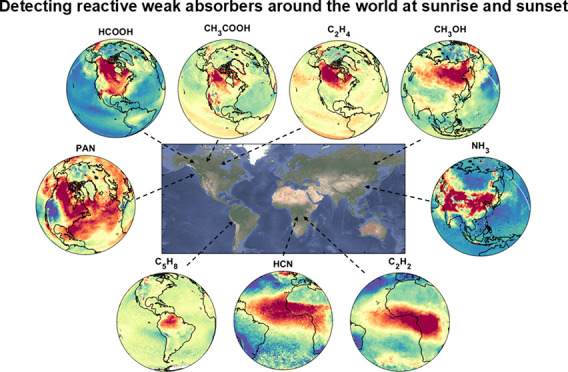

## 1 Introduction

Atmospheric reactive trace gases, such as methane (CH_4_), ozone (O_3_), carbon monoxide (CO), nitrogen oxides, sulfur dioxide, and volatile organic compounds, play vital roles in the atmospheric environment and climate change.^1^ Among them, CH_4_, O_3_, and CO have medium lifetimes ranging from a few weeks to years.^2^ Due to the strong absorption and rich atmospheric abundance, their detection technologies have been relatively well developed, from ground-based instruments to satellite remote sensing, multispectral to hyperspectral and passive optical radiometers or spectrometers to active lidar.^3–6^ The remaining weakly absorbing reactive trace gases are more challenging to detect due to their low atmospheric abundance and shorter lifetimes ranging from a few seconds to days.^7^

Nadir-viewing satellite hyperspectral sounders have made great progress in observing weak absorbers. These sounders can be divided into two categories. One is the UV–vis–NIR sounders with solar radiation as the primary source, such as SCIAMACHY, OMI, GOME-2, TROPOMI, EMI, GEMS, and TEMPO.^8–12^ The other is the thermal infrared (TIR) sounders, the object of this study, with the Earth’s surface emission as the primary source. In the past two decades, with the improvement of instrument radiometric performance and the development of trace gas detection technology, more and more weak absorbers were identified from the TIR sounders aboard operational meteorological satellites (as listed in Table S1).^13^

Currently (as of October 2024), most on-orbit polar operational meteorological satellites are in midmorning (e.g., European Metop-B/C and China’s FY-3D satellites) or afternoon orbits (e.g., American S-NPP/JPSS and China’s FY-3F satellites) with equatorial overpass times at about 9:30 am/pm or 1:30 am/pm (local solar time, LST), respectively.^14^ Although these satellites can provide measurements near the polar region at dawn and dusk hours, they are lacking in most regions, especially at low latitudes. The successful launch of FengYun-3E (FY-3E) on July 5, 2021, complements the lack of measurements of these satellites at dawn and dusk (about 5:30 am/pm LST), which increased the time resolution of global observations to 4 h. Despite carrying TIR sounders, the FY-3 series has not been fully proven to be capable of detecting weak absorbers like other satellites, except for recent work based on traditional methods to detect wildfire-enhanced CO, HCOOH, and PAN from FY-3E/HIRAS-II^15^ and to retrieve NH_3_ from FY-3D/HIRAS.^16^ Therefore, this study expects to confirm that FY-3E/HIRAS-II can identify dozens of weakly absorbing reactive trace gases, just like other similar mature polar-orbiting sounders (including AIRS, TES, IASI, and CrIS, as listed in Table S1),^13,17^ by using state-of-the-art weak absorbers spectral feature quantification (i.e., hyperspectral range index) and identification (i.e., whitening transformation) methods. Additionally, we expect to explore the value of FY-3E/HIRAS-II in advancing our understanding of the diurnal variation of trace gases.

## 2 Methods and Materials

### 2.1 FY-3E

FY-3E/HIRAS-II is the second generation of hyperspectral infrared atmospheric sounder, succeeding the initial FY-3D/HIRAS. HIRAS-II is a Fourier transform spectrometer including three spectral bands: LWIR (650–1168.125 cm^–1^), MWIR (1168.75–1920 cm^–1^), and SWIR (1920.625–2550 cm^–1^), with an unapodized spectral resolution of 0.625 cm^–1^, i.e., 1/2MPD (MPD, maximum optical path difference of 0.8 cm). Its spectral response function approximates the ideal Sinc function, i.e., Sinc­(2MPDσ), where σ is the wavenumber. The scanning mirror works across the track in the general earth observation mode of FY-3E/HIRAS-II (FY-3D/HIRAS). Each cross-track scanning sequence comprises 32 (33) resident fields of regard (FOR), including 28 (29) continuous earth scenes, 2 (2) deep space views, and 2 (2) blackbody observation targets. Compared to FY-3D/HIRAS, the number of detectors in a single resident FOR of FY-3E/HIRAS-II has increased from 2 × 2 to 3 × 3, the nadir ground resolution has increased from 16 to 14 km, and the sensitivity has increased by more than 2 times.^18,19^ The noise equivalent temperature difference of FY-3E/HIRAS-II in the spectral region used here (∼700–1900 cm^–1^) is within 0.2 and 0.4 K, as referenced from the L1 product instruction document (as in the Data Availability Statement).

### 2.2 Forward Model

The line-by-line radiative transfer model (LBLRTM) is used as a forward model to calculate the spectral sensitivity of HIRAS-II to the variations of each gas column. LBLRTM is an open-access, accurate radiative transfer model that can be used for radiation simulations in the ultraviolet to microwave spectral region, providing the basis for many radiative transfer applications, including trace gas detection and retrieval.^20–22^ LBLRTM uses molecular absorption spectroscopic line parameters from the HITRAN database and other additional line parameters.^23,24^ Note that the absorption spectroscopic line parameters of HONO are not included in LBLRTM and need to be downloaded from HITRAN (https://hitran.org/). These line parameters are extracted by LNFL (a line file creation program) for use in LBLRTM. The MT_CKD continuum database is also used in LBLRTM to account for the H_2_O, CO_2_, O_2_, and N_2_ continuum in the thermal infrared.^25^ The sensitivity (i.e., Jacobian, *K*) of HIRAS-II to changes in the gas column can be calculated as eq 1. Here, BT is the LBLRTM-simulated HIRAS-II brightness temperature. Figure S1 shows the calculated Jacobian values of each gas.

1 $$K_{\mathrm{gas}}=\frac{\partial \mathrm{BT}}{\partial {\mathrm{column}}_{\mathrm{gas}}}$$

### 2.3 Hyperspectral Range Index and Whitening Transformation

The detection methods for weak absorbers have evolved from the traditional spectral fitting method based on optimal estimation to the current techniques called hyperspectral range index (HRI)^26,27^ and whitening transformation,^13^ as listed in Table S1. HRI is calculated as in eq 2, quantifying the spectral vector (*y*, BT) within a continuous spectral interval containing the target gas’s sensitive channels into a single number representing its spectral contribution.

$$\mathrm{HRI}=\frac{K_{\mathrm{gas}}^{T}{S_{y}}^{-1}\left(y-\mathrm{y̅}\right)}{\sqrt{K_{\mathrm{gas}}^{T}{S_{y}}^{-1}K_{\mathrm{gas}}}}\frac{1}{N}$$ 2

HRI relies on the average spectral vector *y̅* and its associated covariance matrix *S*_*y*_ calculated from carefully selected background spectra (Text S1). The pair {*y̅*, *S*_*y*_} describes the spectra distribution and how they vary under different surface-atmosphere thermodynamic parameters and atmospheric composition related to the background spectra.^13^ As shown in Figure S2a, by construction, the HRIs of the background spectra are Gaussian distributed with a mean of zero and a standard deviation of one. Thus, a HIRAS-II spectrum with an HRI absolute value exceeding the mean (i.e., zero) by 1, 2, and 4 is considered to have a target gas enhancement with statistical significances of 68.27%, 95.45%, and 99.99%, respectively.

HRI is a sensitive spectral index but might be prone to false detection when a major spectral interference (e.g., another trace gas, water vapor, or unusual surface emissivity) overlaps with the absorption band of the target gas.^28,29^ De Longueville et al.^13^ introduced a complementary technique called whitening transformation, which is naturally related to HRI and can be used to interpret or verify whether the spectral contribution comes from the target gas. Specifically, the formula of HRI was originally derived from the least-squares estimation, where the covariance matrix can be explained as a generalized noise covariance matrix and all background variation is considered noise. In the field of hyperspectral imagery, the covariance matrix is properly called the background clutter matrix, and the HRI is called the signal-to-clutter or signal-to-noise ratio, and its expression can sometimes be written as^30^

3 $$\mathrm{HRI}=\frac{K_{\mathrm{gas},\mathrm{whitened}}^{T}y_{\mathrm{whitened}}}{||K_{\mathrm{gas},\mathrm{whitened}}||}$$

with

4 $$K_{\mathrm{gas},\mathrm{whitened}}={S_{y}}^{-1/2}K_{\mathrm{gas}}$$

and

5 $$y_{\mathrm{whitened}}={S_{y}}^{-1/2}\left(y-\mathrm{y̅}\right)$$

Equation 5 removes most of the background signal by subtracting the background spectrum from the measured spectrum and assigning the variability of the background spectrum as a weight. By transformation, the spectrum of each channel in the background spectra is converted to uncorrelated random variables with an ideal zero mean and a unit standard deviation (Figure S2b). This operation transforms generalized noise into white noise, so it is called whitening.^30,31^ Therefore, when a spectrum is whitened and some of the channel abnormalities deviate from the mean by more than one standard deviation, it is suspected that there is a contribution(s) from one or more absorbers. By comparing the whitened spectrum with the whitened Jacobian (eq 4) of potential absorbers, it is possible to determine whether the spectral anomalies come from the target gas by visual inspection or, here, by quantifying the correlation coefficient between the two, as shown in the case of Figure S2c.

## 3 Results and Discussion

### 3.1 Identifying 14 Weakly Absorbing Reactive Gases

Figure 1 presents 14 weak absorbers identified from the FY-3E/HIRAS-II measurements. We identified eight weak absorbers in a single measurement over a wildfire in Canada on July 10, 2023 (Figure 1b). C_2_H_4_ has the strongest signal in this whitened spectrum, with a value of −12.74 at 949.375 cm^–1^ and several other features on both sides. NH_3_ has multiple sensitive channels in the broad region from 812 to 1126 cm^–1^, among which the two channels with the strongest spectral signals are located on the left and right sides of C_2_H_4_, at 931.25 and 966.875 cm^–1^, respectively. Strong Q-branches of HCN and C_2_H_2_ and several R lines are observed between 700 and 800 cm^–1^. The most sensitive channels for HCN and C_2_H_2_ are 712.5 and 729.375 cm^–1^, respectively. We observe the absorption peaks of CH_3_OH and HCOOH at around 1033.75 and 1105 cm^–1^, respectively. The broadband absorbers PAN and CH_3_COOH are observed between 760 and 900 cm^–1^ and between 1120 and 1238.75 cm^–1^, respectively. The above eight gases and C_5_H_8_ identified from a spectrum over the Amazon (Figure 1c) are the routine weak absorbers defined in this study (Section 3.2). In other measurements this month, we also identified HONO, C_4_H_4_O, SO_2_, C_3_H_6_, and H_2_CO. These five gases have weak spectral features and can be detected in only a few measurements; therefore, they are not included in the routine gases.

**Figure 1:**
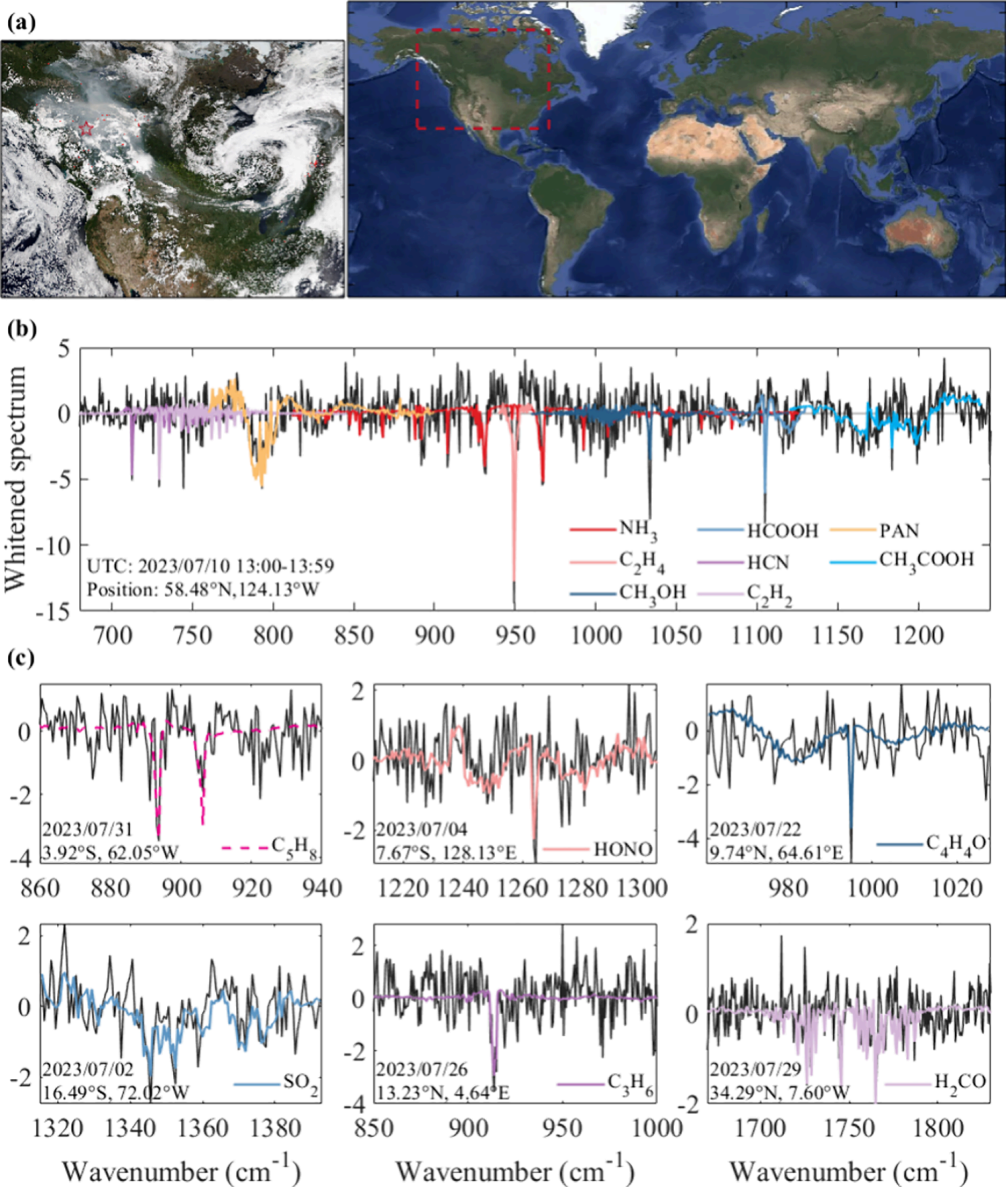
Fourteen weakly absorbing reactive trace gases identified from July 2023 FY-3E/HIRAS-II clear-sky measurements. (a) Global satellite true-color imagery, obtained from Google Map, Imagery Copyright 2024 NASA. The left panel plots the NOAA-20/VIIRS visible imagery of North America on July 10, 2023, acquired from NASA Worldview. (b) Eight weakly absorbing reactive gases identified in a spectrum over biomass burning, as marked by the red star in the left panel of (a). The black line represents the whitened spectrum, and the colored lines represent the whitened Jacobian spectrum of each gas. Note that these whitened Jacobians are appropriately scaled to match the whitened spectrum. (c) Identification of the remaining six weak absorbers. We annotate the date and location of the spectral measurement in each subfigure. The screening method for FY-3E/HIRAS-II clear-sky measurements is described in Text S2.

### 3.2 Global Spectral Features of Nine Routine Weakly Absorbing Reactive Gases

Figure 2 plots the global monthly average distribution of the postfiltered (Text S3) HRI of nine routine weak absorbers. Here, “routine” refers to gases with extensive anthropogenic and natural sources or abundant precursors, allowing us to derive reliable spectral features globally. The spectral features of these gases show different spatial distributions related to emission sources, gas lifetime, atmospheric abundance, and instrument sensitivity. Here, we observe significant spectral enhancements of almost all gases over North America due to the severe wildfires in the Canadian boreal forest and their transport.^32^ We also capture enhanced C_2_H_2_, HCN, CH_3_OH, HCOOH, and PAN in the African rainforest, as well as enhanced CH_3_OH, HCOOH, and PAN in East Siberia and other regions with intensive biomass-burning activities. These findings are consistent with satellite observations over the past years.^17,33–35^

**Figure 2:**
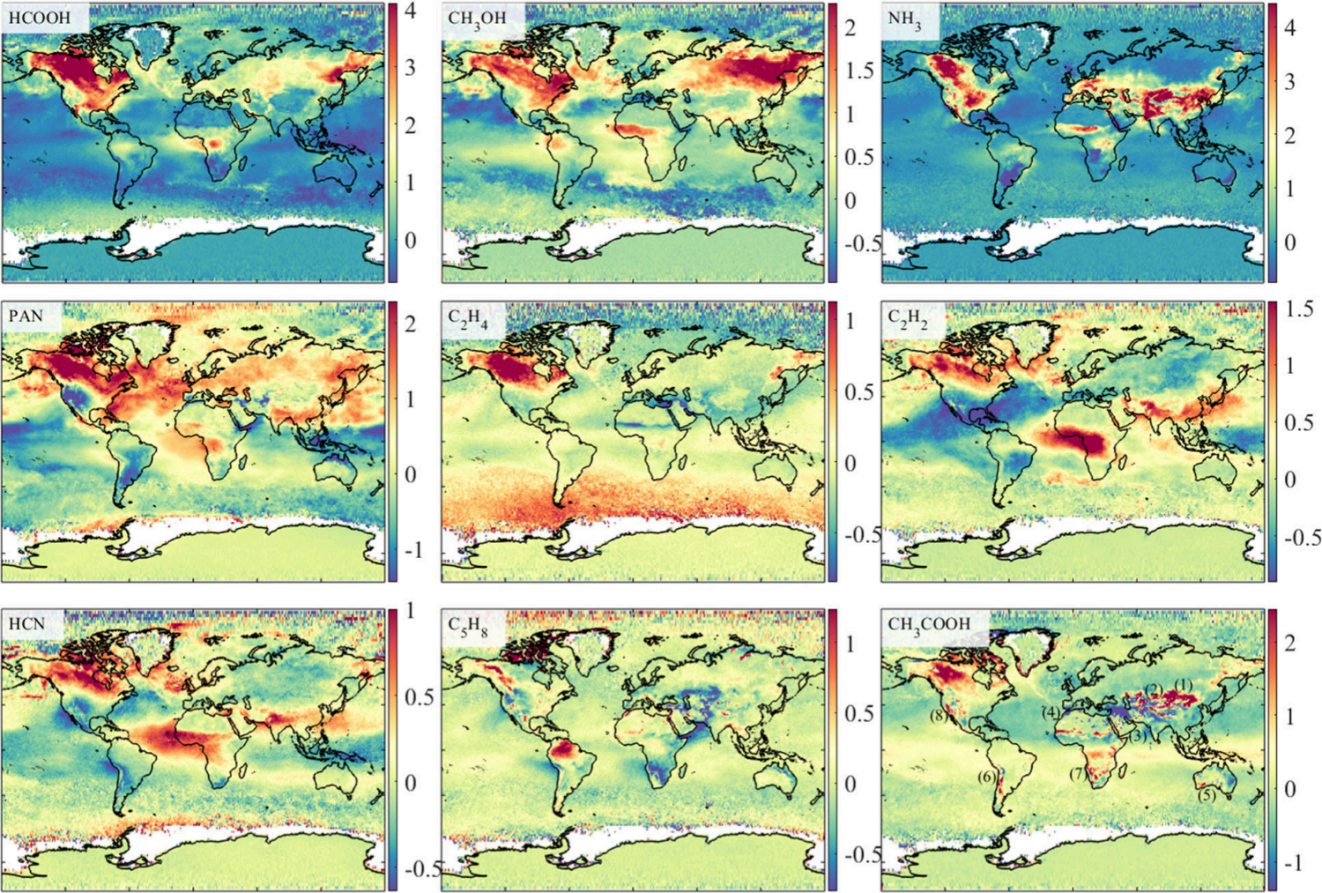
Global distribution of the postfiltered spectral signatures (i.e., HRI) of nine routine weakly absorbing reactive trace gases plotted at 0.5 × 0.5°, calculated from more than 35.4 million FY-3E/HIRAS-II clear-sky measurements in July 2023. We have marked eight typical desert regions in the last panel, numbered from (1) to (8): Gobi Desert, Taklamakan Desert, Arabian Desert, Saharan Desert, Great Australian Desert, Atacama Desert, Namib Desert, and Mojave Desert. Here, the positive and negative values of HRI are partially related to thermal contrast, and an HRI with an absolute value less than 1 usually corresponds to the background concentration level.

In areas with intensive human activity, such as central China and northern India, we observe strong NH_3_ signals consistent with previous satellite observations.^36^ Here, the NH_3_ HRI in western China is even larger than that in the North China Plain, which is the main source of NH_3_ in China. This is partly due to the large thermal contrast in western China (Figure S7), which provides favorable observation conditions and improves the instrument sensitivity. The relationship between the column, HRI, and thermal contrast (TC) plotted in Figure 3d provides a more detailed explanation. In tropical rainforests such as the Amazon, we observe biogenic isoprene signatures above background levels consistent with recent satellite retrievals.^37–39^ Franco et al.^34^ showed that the atmospheric HCOOH and CH_3_COOH columns caused by wildfires should have similar spatial patterns, whereas their spectral features here have different spatial patterns. It is partly due to the complex nonlinear relationship between the gas column and its corresponding spectral feature, which is affected by surface-atmospheric parameters and satellite observation geometry. Furthermore, CH_3_COOH is more prone to interference from surface emissivity and residual clouds than HCOOH,^34^ which may be the reason for the abnormal enhancement of CH_3_COOH HRI over the Himalayas and Gobi Desert in Figure 2. The HRI value is usually positively correlated with its corresponding column when the interfering parameters remain unchanged for optically thin reactive trace gases.^40^ Therefore, temporally persistent HRI enhancements can be used to identify emitters of these reactive trace gases.^29,41–43^

**Figure 3:**
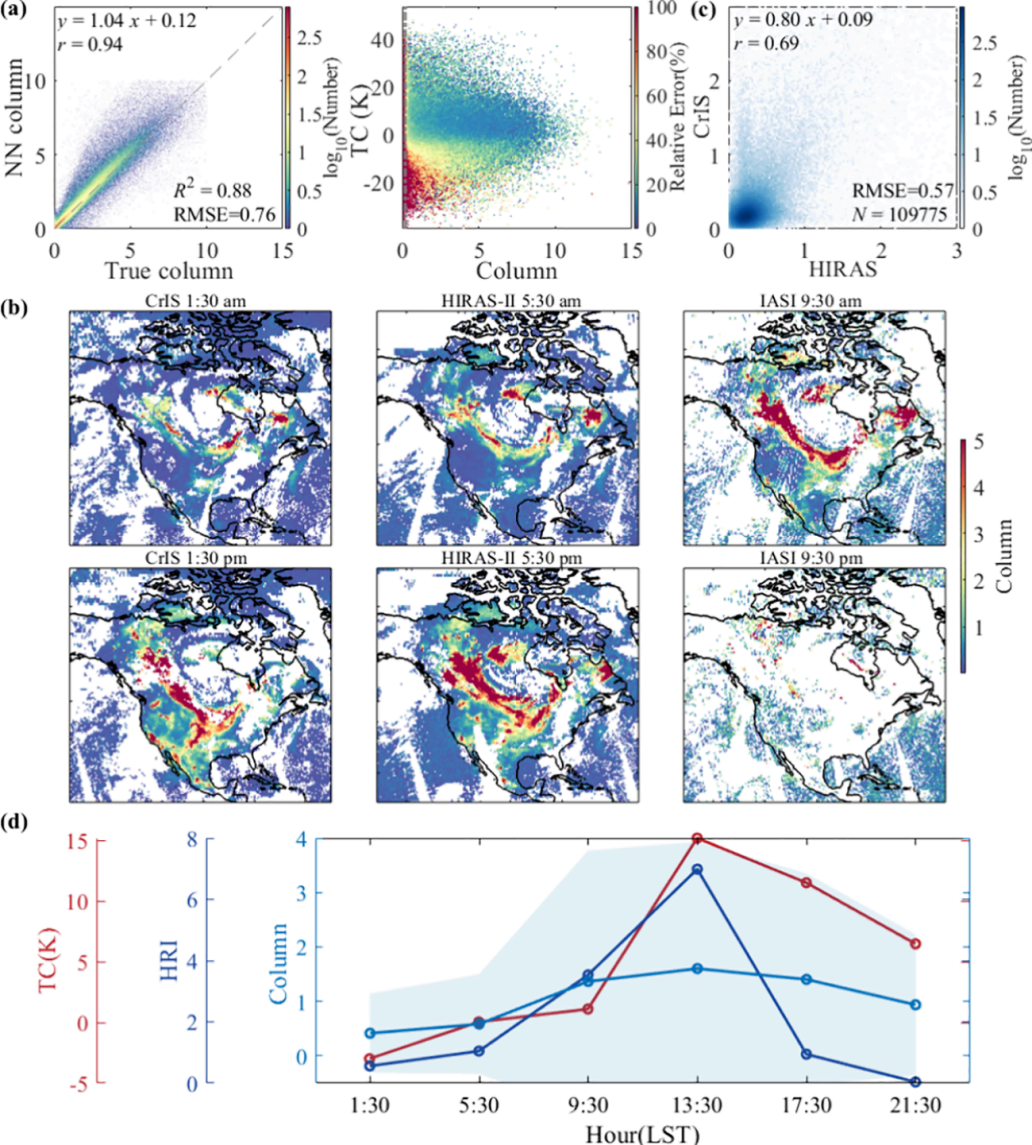
Retrieval performance and diurnal variation of the NH_3_ column. (a) Relationship between the true column and the NN-retrieved column (× 10^16^ molecules/cm^2^), and the relative uncertainty of the NN model in the unit column and TC grid, respectively. The gray dotted line in the right panel represents the marking lines for the column of 0.2 × 10^16^ molecules/cm^2^. (b) Diurnal variation of the NH_3_ over wildfires on July 15, 2023, composed of FY-3E/HIRAS-II, SNPP/CrIS, and Metop-B/IASI. (c) Relationship between HIRAS-II and CrIS NH_3_ grid-averaged global columns on July 15, 2023. The relationship between HIRAS-II and IASI is shown in Figure S9. (d) Diurnal variations of the regionally averaged NH_3_ column, HRI, and TC. The shaded area represents one standard deviation of the NH_3_ column. Here, we match data from the three sounders within the unit grid (0.5 × 0.5°) over the entire area (b) and then calculate the regional averages. “Match” refers to selecting the retrievals where all three sounders have valid values, except for the IASI at nighttime, which is too sparse.

### 3.3 Converting HRI into Columns: The Case of Ammonia in Wildfires

Referring to the HRI-driven neural network (NN) retrieval technique (Text S4),^35,40,44–46^ we converted the NH_3_ HRI into columns to further explore the value of FY-3E/HIRAS-II. Figure 3a shows that the NN model trained for NH_3_ reproduces 88% of the column variance in the training set with an RMSE of 0.76 × 10^16^ molecules/cm^2^. When the column is larger than 0.2 × 10^16^ molecules/cm^2^, the relative error of the model is usually less than 20%, and the relative error is larger when the thermal contrast is negative. We converted the NH_3_ HRI calculated from FY-3E/HIRAS-II and SNPP/CrIS (Figure S8) into columns using the NN model trained above. Hence, the column differences between HIRAS-II and CrIS are only due to the instrument’s radiometric performance and overpass time. The IASI columns plotted in Figure 3b are taken from the official Metop-B/IASI NN product.^40^ The retrieval details we adopted here, such as the parametrization scheme of the NH_3_ profile and the postfiltering of the column, are the same as those in the IASI method to reduce additional biases from the NN models. As shown in Figure 3c, the correlation coefficients between HIRAS-II and the CrIS and IASI columns are 0.69 and 0.66, respectively, and the RMSEs are 0.57 and 1.15 × 10^16^ molecules/cm^2^, respectively.

Figure 3b plots the diurnal variation of the NH_3_ column over the wildfire after prefiltering and postfiltering. Overall, the NH_3_ columns of the three sounders show a consistent spatial distribution. Figure 3d shows that the differences between the surface and air temperatures (TC, thermal contrast) are small at night and dawn, varying between −2.99 and 0.05 K. The maximum difference reaches 15.19 K at 13:30, thus providing the most favorable measurement conditions. The columns retrieved by CrIS in the afternoon (13:30) are 3.8 times those retrieved at midnight (1:30). The columns retrieved by HIRAS-II at dusk are 1.8 times those retrieved at dawn, and the columns retrieved by IASI in the morning are 1.5 times those observed in the evening. The diurnal variations of HRI and column are consistent with those of TC.

This study fully explored the potential of FY-3E/HIRAS-II in detecting reactive weak absorbers. The ammonia column retrieval over extreme wildfires in North America demonstrates that FY-3E/HIRAS-II can enhance our understanding of the diurnal variation of trace gases by complementing measurements at dawn and dusk. However, further efforts are necessary to improve consistency between products from different sounders and reduce the bias introduced by retrieval technology to assess this diurnal variation more accurately. It is foreseeable that FY-3E/HIRAS-II would be particularly valuable for tracking the transport and evolution of short-lived reactive trace gases in extreme events.

## Supplementary Material



## Data Availability

The FY-3E data are available at https://satellite.nsmc.org.cn/PortalSite/Data/Satellite.aspx. The FY-3E/HIRAS-II L1 product instruction document is available at http://satellite.nsmc.org.cn/DataPortal/cn/support/document.html?TypeID=1100#guide-fy3e. Detailed instructions for the LBLRTM and running examples are available at https://github.com/AER-RC/LBLRTM.

## Notes

The history and technology of weakly absorbing reactive trace gas detection; details on FY-3E/HIRAS-II background spectra selection and cloud screen; postfiltering of the spectral features of each gas and the technique for the ammonia column retrieval; Jacobians of weakly absorbing reactive trace gases; performance of hyperspectral range index and whitening transformation; global distribution of spectral signatures of routine weakly absorbing reactive trace gases; normalized frequency distributions of HRI; mask of deserts and polar regions; spatial distribution of monthly mean TCs in China; comparison of NH_3_ HRIs; and relationship between HIRAS-II and IASI NH_3_ grid-averaged global columns (PDF)
