# Supplementary material for: Advancing Atmospheric Detection of Weakly Absorbing Reactive Trace Gases Using the FY-3E/HIRAS-II TIR Sounder on a Dawn–Dusk Orbit
Source: Environ Sci Technol Lett. 2025 Jun 24;12(7):848–55. doi: 10.1021/acs.estlett.5c00501 (PMC12243120; doi:10.1021/acs.estlett.5c00501)
Supplement: Supplementary file 1 [file ez5c00501_si_001.pdf]

Supporting Information for

Advancing Atmospheric Detection of Weakly Absorbing Reactive Trace Gases Using the FY-3E/HIRAS-II TIR Sounder on a Dawn-Dusk Orbit

Zhenxing Liang[1], Dasa Gu[1,2 *], Rui Li[3], Jian Liu[4], Chengxing Zhai[5], Hui Su[6], Alexis K. H. Lau[1,6]

[1]Division of Environment and Sustainability, The Hong Kong University of Science and Technology, Clear Water Bay, Hong Kong SAR 999077, China

[2]Guangdong-Hongkong-Macau Joint Laboratory of Collaborative Innovation for Environmental Quality, The Hong Kong University of Science and Technology, Clear Water Bay, Hong Kong SAR 999077, China

[3]School of Earth and Space Science, CMA-USTC Laboratory of Fengyun Remote Sensing, Deep Space Exploration Laboratory, University of Science and Technology of China, Hefei 230026, China

[4]College of Environment and Ecology, Shanxi Key Laboratory of Complex Air Pollution Control and Carbon Reduction, Taiyuan University of Technology, Taiyuan 030024, China

[5]Division of Emerging Interdisciplinary Areas, The Hong Kong University of Science and Technology, Clear Water Bay, Hong Kong SAR 999077, China

[6]Department of Civil and Environmental Engineering, The Hong Kong University of Science and Technology, Clear Water Bay, Hong Kong SAR 999077, China

[*]*Corresponding author: Dasa Gu*, Phone: (852) 23359317; Email: *dasagu@ust.hk*

Contents of this file

Table S1

Text S1 to Text S4

Figure S1 to Figure S9

Table S1

Table. Weakly absorbing reactive trace gases detected by the Nadir TIR Sounders.

Year	Species	Scene	Detection method	Satellite/Sensor	Reference
2005	*Sulfur dioxide* (SO_2)	Volcanic eruption	Spectral fitting method	Aqua/AIRS	Carn et al.[1]
2008	***Ammonia*** (NH_3)<br>***Methanol*** (CH_3OH)	Megacity	Spectral fitting method	Aura/TES	Beer et al.[2]
2009	***Ethylene*** (C_2H_4)<br>***Formic acid*** (HCOOH)<br>***Peroxyacetyl nitrate*** (PAN)	Biomass burning	Spectral fitting method	MetOp/IASI	Coheur et al.[3]
2011	***Hydrogen cyanide*** (HCN)<br>***Acetylene*** (C_2H_2)<br>***Acetic acid*** (CH_3COOH)<br>*Nitrous acid* (HONO)<br>*Furan* (C_4H_4O)<br>*Formaldehyde* (HCHO)<br>*Propylene* (C_3H_6)	Biomass burning	Spectral fitting method	MetOp/IASI	Clarisse et al.[4]
2011	Hydrogen sulfide (H_2S)	Volcanic eruption	Spectral fitting method	MetOp/IASI	Clarisse et al.[5]
2019	***Isoprene*** (C_5H_8)	Amazon	Spectral fitting method	SNPP/CrIS	Fu et al.[6]
2021	Glycolaldehyde (HOCH2CHO)	Biomass burning	Hyperspectral range index & Whitening Transformation	MetOp/IASI	De Longueville et al.[7]

Table S1 lists these weak absorbers, corresponding sounders, detection technologies, and scenes according to the time order. Note that the definition of reactive trace gases mainly refers to Clarisse et al.[4]. The gases successfully identified from FY-3E/HIRAS-II in this study are indicated in italics, and the gases whose spectral features can be reliably mapped on a global scale are indicated in bold.

Text S1. The selection of background spectra

There is no enhancement of the target gas in the background spectra; that is, no target gas appears or corresponds only to its background concentration level. The selection process of representative background spectra of HIRAS-II in this study is mainly divided into two steps. The first step is to perform regular spatiotemporal sampling of HIRAS-II measurements to obtain broadly representative spectra rather than over-representing a specific region. The second step is to perform iterative filtering on these representative spectra to extract the background spectra. The iterative filtering process for gas is as follows: First, the initial HRIs of all spectra are calculated, those spectrums exceeding the HRI threshold (here, 2) are filtered out, and then the updated HRIs of the remaining spectrums are calculated until the iteration converges to spectra set less than the HRI threshold, which is the background spectra. Note that in each process, the scaling factor N in the HRI calculation equation is taken from the average of HRIs where no target gas appears over the remote ocean. The role of the normalization factor N is to guarantee that the measured spectra corresponding to the HRIs with a standard deviation of 1 contain only the target gas within the background concentration levels.

Text S2. FY-3E/HIRAS-II and SNPP/CrIS cloud screen

Pre-filtering removes those measurements with erroneous L1 or excess cloud coverage. The radiation properties of the cloud and its blocking of radiation below make it impossible for spaceborne passive infrared sounders to measure the trace gases in and below clouds, so this study only considers FY-3E/HIRAS-II measurements under cloud-free conditions. The cloud mask (CLM) product from the Medium Resolution Spectral Imager (MERSI)[8], also onboard FY-3E, is used to screen clear-sky measurements. Specifically, we matched the surrounding CLM products

for each FY-3E/HIRAS-II footprint and kept FY-3E/HIRAS-II measurements with at least 80% of the CLMs marked as clear or probably clear. The cloud screen operation of SNPP/CrIS is consistent with that of HIRAS, and the cloud masks corresponding to the SNPP/CrIS coverage are directly taken from the SNPP CrIS IMG product (https://disc.gsfc.nasa.gov/datasets/SNDRSNCrISL1BIMG_2/summary).

Text S3. The post-filtering mechanism of spectral features and its performance

As described in the second paragraph of Section 2.3, a HIRAS-II spectrum with an HRI absolute value exceeding the mean (i.e., zero) by 1, 2, 3, and 4 is considered to have a target gas enhancement with a statistical significance of 68.27%, 95.45%, 99.73%, and 99.99%, respectively. In the monthly average distribution of the original spectral features without post-filtering (Figure S3), we observed many abnormal spectral features due to the contamination of the whitened spectrum by unusual surface emissivity, especially for broadband absorbers like PAN and CH_3COOH. For example, there are many measurements with HRI greater than 1 in HCOOH, NH_3, PAN, C_2H_4, C_5H_8, and CH_3COOH over the Arabian Desert, Saharan Desert, and Great Australian Desert. We also observed abnormal CH_3COOH over Antarctica, which may be caused by low instrument sensitivity due to low temperatures.

As also stated in the fourth paragraph of Section 2.3, by comparing the whitened spectrum with the whitened Jacobian, it is possible to determine whether the spectral anomalies come from the target gas by visual inspection or, here, by quantifying the correlation coefficient between the two, as shown in the case of Figure S2(c). That is, the higher the correlation coefficient between the whitened spectrum and Jacobian, the more likely the spectral anomaly is from real target enhancement. Therefore, to filter out those unrealistic spectral features over deserts and polar

regions, we counted the frequency distribution of the HRI (blue plot in Figure S4) and the frequency distribution of the correlation coefficient between its whitened spectrum and Jacobian (blue plot in Figure S5) corresponding to more than 7.40 million measurements over the desert and polar regions in that month. We also counted the information above the hotspots (purple plots in Figure S4 and Figure S5) to facilitate observation and set post-filtering thresholds. The masks of the desert and polar regions are based on the MODIS land cover product (shrubs and unvegetated regions in MCD12C1), as shown in Figure S6. For isoprene, we chose the Amazon rainforest as the hotspot area, and for the other gases, we chose North America as the hotspot area.

As shown in Figure S4, over deserts and polar regions, the HRIs of all gases follow the Gaussian distributions with mean values close to 0, which is similar to random noise and close to the HRI distribution of background measurements. Over hotspots, due to the real enhancement of target gases, the frequency distribution of HRI of each gas deviates from the above Gaussian distribution, and the degree of deviation is related to the number of measurements with target enhancement. As shown in Figure S3, the HRI of HCOOH is generally large in North America, while the HRI of C_5H_8 is relatively small in the Amazon region. Therefore, in the frequency distribution, the degree of deviation of C_5H_8 from the Gaussian distribution is less than that of HCOOH. In Figure S5, the frequency distribution of the correlation coefficient between the whitened spectrum and Jacobian of each gas is consistent with the performance of HRI.

Based on the above analysis, we set the following two unified post-filtering mechanisms for each gas: (1) Over deserts and polar regions, if the HRI of the gas is greater than 1, but the correlation coefficient between its corresponding whitened spectrum and Jacobian is less than 0.6 or the threshold, it will be filtered out. Here, the threshold of the correlation coefficient is calculated as its mean value plus three times the standard deviation. (2) Over deserts and polar

regions, if the HRI of the gas is larger than the threshold, it will also be filtered out. Here, the threshold is calculated as the mean HRI over the hotspot area plus three times the standard deviation. This post-filtering mechanism filters out more than 50% (ranging from 50.49% to 78.70% for nine gases) of the measurements over deserts and polar regions. These filtered measurements have unrealistic abnormal HRI values. The spatial distribution of spectral features after filtering (Fig. 2 in the main text) shows that the abnormal CH_3COOH values in the Antarctic region and most of the abnormal HRI values in the desert region are filtered out, such as C_5H_8, PAN, HCOOH, NH_3, and CH_3COOH.

Text S4. HRI-driven neural network (NN) retrieval technique

Converting the spectral features into columns is another key technology to further exploit the value of FY-3E/HIRAS-II. The two commonly used methods are the optimal estimation (OE) based physical method[9] and the HRI-driven machine learning (ML) method[10]. OE relies on an iterative process of fitting satellite-measured radiances with radiative transfer model-simulated radiances, which requires intensive computing resources. The efficient HRI-driven ML method (called ANNI, Artificial Neural Network for IASI) was proposed by Whitburn et al.[10] to convert the HRI of NH_3 calculated from IASI into the atmospheric total column and has been gradually improved in the past few years[11–13], and widely used for the retrieval of other sounders and weak absorbers[14–22]. Such a method mainly includes two steps: (1) calculating the HRIs of the target gas from the satellite-measured radiances and (2) converting the above HRIs into columns based on a carefully trained ML model. Training this ML model relies on widely representative simulated training sets.

Specifically, we simulate more than 500,000 radiances received by FY-3E/HIRAS-II under different surface-atmospheric parameters, satellite observation geometries and target gas columns through the accurate line-by-line radiative transfer model (LBLRTM, as in Section 2.2). Various target gas columns are generated by randomly scaling the vertical reference profile of the target gas. The shape of the NH_3 vertical reference profile is characterized by the Gaussian parameterization scheme, same in ANNI V4[11]. Then, we calculate the HRIs of the target gas from the LBLRTM-simulated radiances. Finally, based on the ML method, we train the nonlinear relationship between the above target gas column (model output) and the corresponding HRI and auxiliary parameters (model input). The auxiliary parameters include thermal contrast, surface temperature, pressure, atmospheric temperature, and water vapor column, which are taken from the ERA5 (the fifth generation ECMWF reanalysis for global climate and weather) data. Two Gaussian function parameters, which respectively characterize the layer height and layer thickness of the vertical profile shape of NH_3, are also used as auxiliary parameters. The ML model refers to previous studies and adopts an artificial neural network (NN) with two hidden layers (here, 20 and 10 nodes, respectively) with a hyperbolic sigmoid transfer function and a linear transfer function in the output layer.

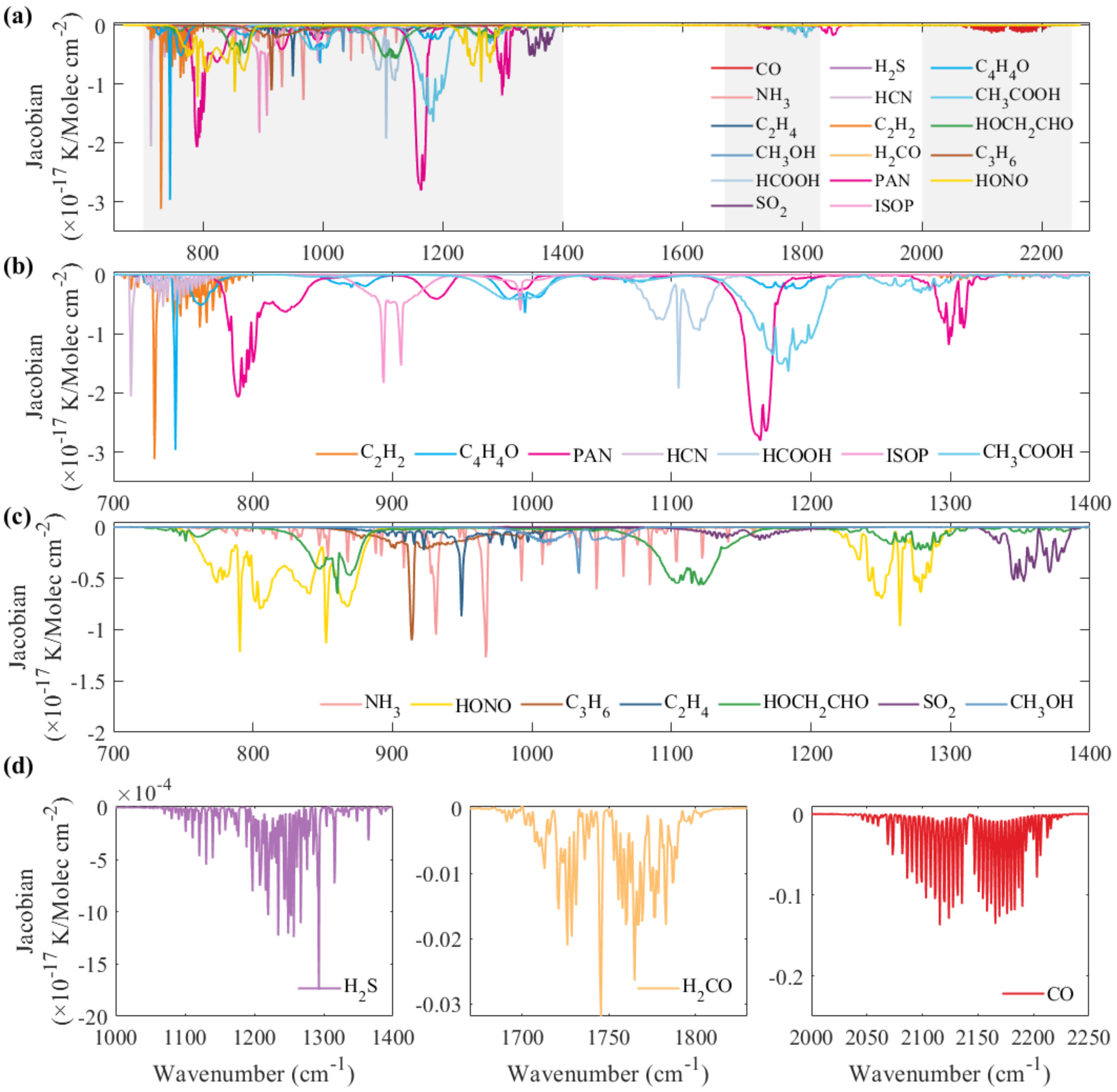

Figure S1. (a) Jacobian of CO and weakly absorbing reactive trace gases calculated in the continuous spectral interval 650-2250 cm^{-1}. The three gray-masked intervals indicate the presence of target absorption features. (b) and (c) plot the 14 reactive trace gases in the first mask interval 700-1400 cm^{-1} in order according to the Jacobian maximum value. Within this interval, the sensitivity of hydrogen sulfide (H_2S) is too weak and is plotted alone in the left panel of (d). The middle panel of (d) plots the Jacobian of formaldehyde (H_2CO), which absorbs weakly in the second mask interval 1670-1830 cm^{-1}. The right panel of (d) plots the Jacobian

of the strong absorber CO in the third interval 2000-2250 cm^{-1}. Note: The Jacobian alone represents only the instrument's sensitivity to changes in the gas column; the specific spectral contribution the gas produces also depends on its atmospheric abundance. The trace gas profiles used in the radiation simulation are taken from the built-in standard climatological profiles in LBLRTM. The LBLRTM-simulated radiance is convolved with the spectral response function of HIRAS-II to characterize the instrument measurements.

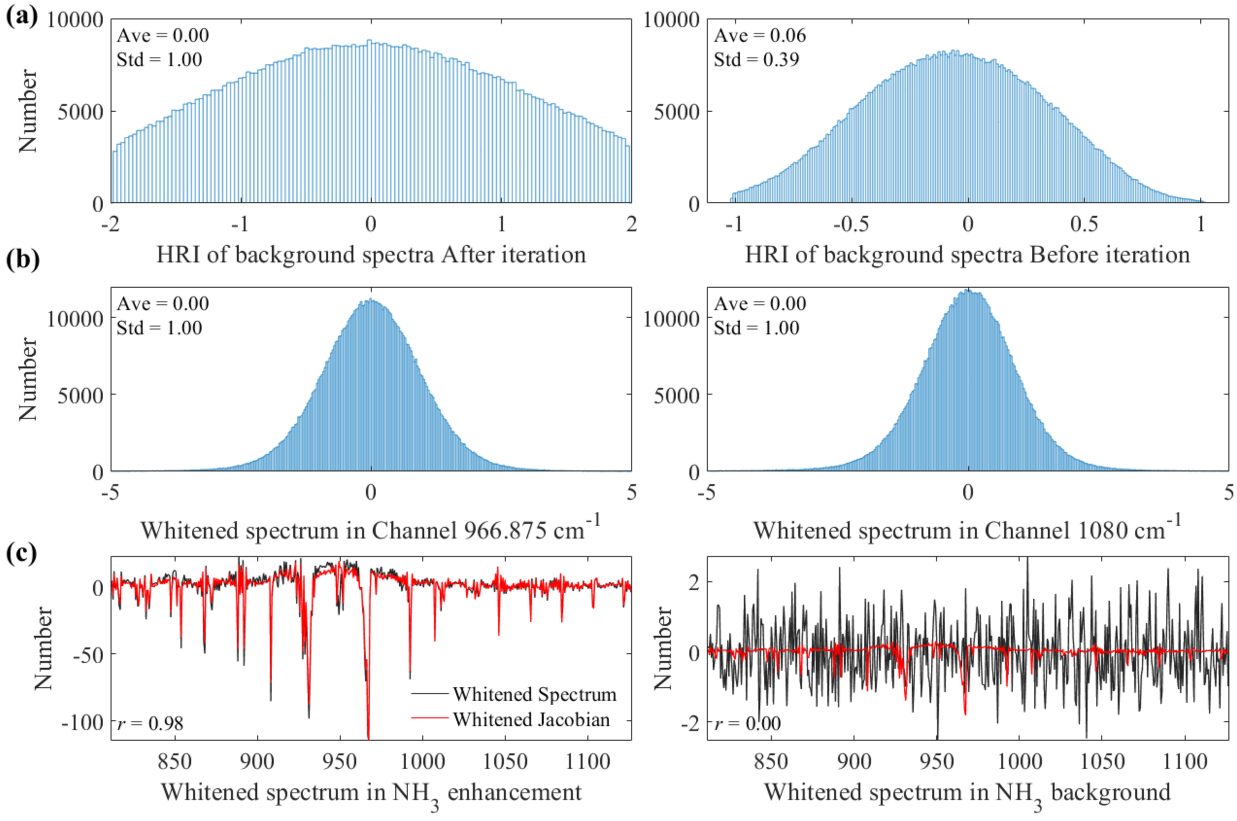

Figure S2. Hyperspectral range index and whitening transformation, using ammonia as an example. (a) Left panel: frequency distribution of HRI of the iteratively selected background spectra; Right panel: original HRI of the background spectra, where the average spectral vector and associated covariance used in the HRI calculation are taken from all representative spectra, which average and standard deviation deviate from 0 and 1, respectively. (b) Left panel: distribution of whitened background spectra at an NH_3-sensitive channel (966.875 cm^{-1}); Right panel: distribution of whitened background spectra at a non-NH_3-sensitive channel (1080 cm^{-1}). Whether it is an NH_3-sensitive channel or a non-sensitive channel, the background spectra of each channel after whitening have a mean of zero and a standard deviation of 1. (c) Left panel: the whitened spectrum and the whitened Jacobian in a spectrum enhanced by NH3, which are highly correlated with a correlation coefficient of 0.98; Right panel: the whitened spectrum and the whitened Jacobian in a background spectrum. Note that the whitened Jacobian is scaled to match the whitened spectrum.

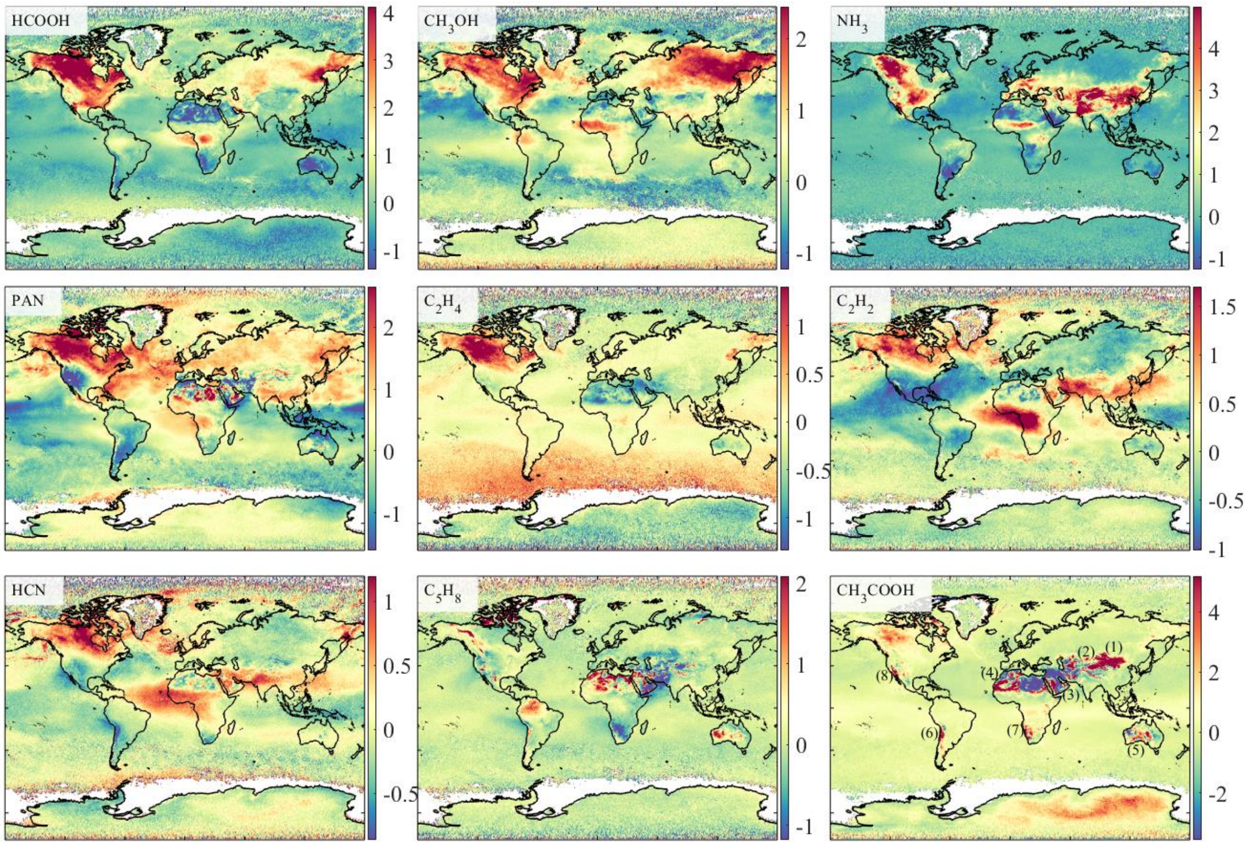

Figure S3. Global distribution of the spectral signatures (i.e., HRI) of nine routine weakly absorbing reactive trace gases plotted at 0.5×0.5°, calculated from more than 35.4 million FY-3E/HIRAS-II clear-sky measurements in July 2023. We have marked eight typical desert regions in the last panel, numbered from (1) to (8): Gobi Desert, Taklamakan Desert, Arabian Desert, Saharan Desert, Great Australian Desert, Atacama Desert, Namib Desert and Mojave Desert. The positive and negative values of HRI are partially related to thermal contrast, and HRI with an absolute value less than 1 usually corresponds to the background concentration level.

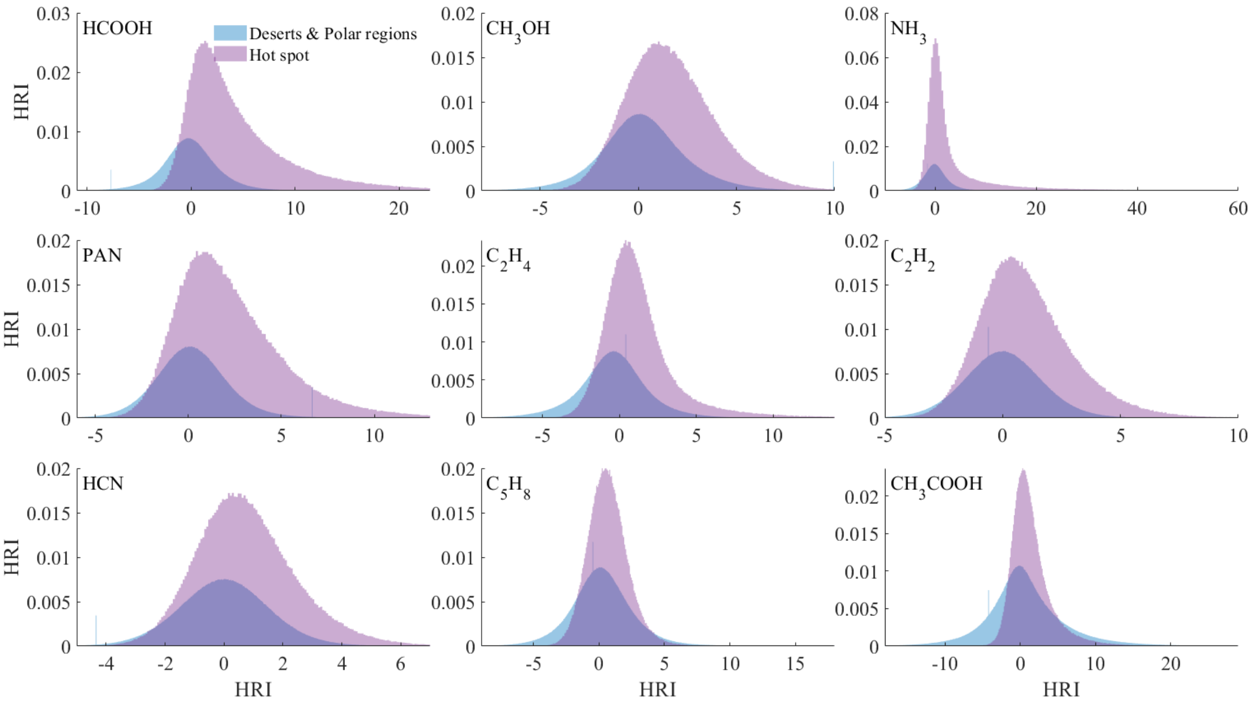

Figure S4. Normalized frequency distributions of HRI corresponding to FY-3E/HIRAS-II measurements over specific areas in July 2023. The purple plots represent the distribution over hotspots, and the blue represents deserts and polar regions.

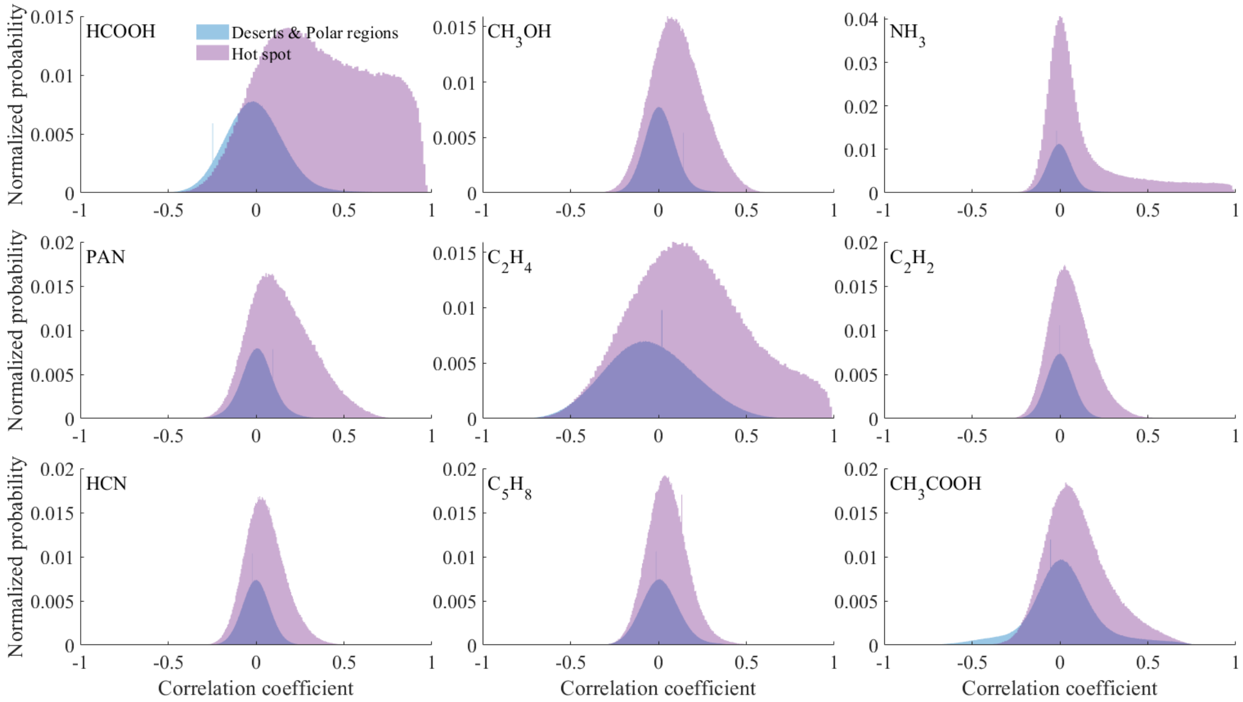

Figure S5. Normalized frequency distributions of the correlation coefficient between the whitened spectrum and Jacobian corresponding to FY-3E/HIRAS-II measurements over specific areas in July 2023. The purple plots represent the distribution over hotspots, and the blue represents deserts and polar regions.

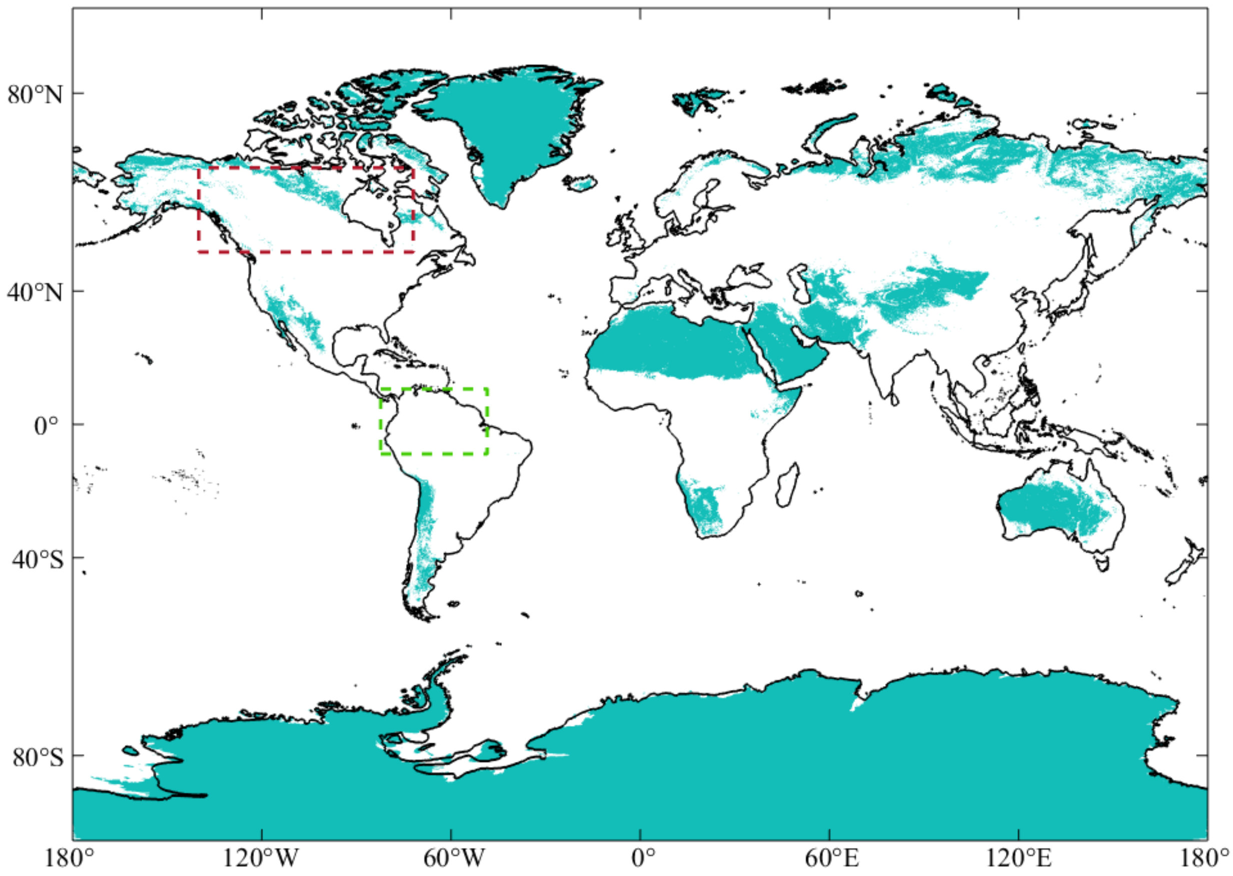

Figure S6. Mask of deserts and polar regions based on the MODIS land cover product. Hotspots are outlined in red (for gases other than isoprene) and green (for isoprene).

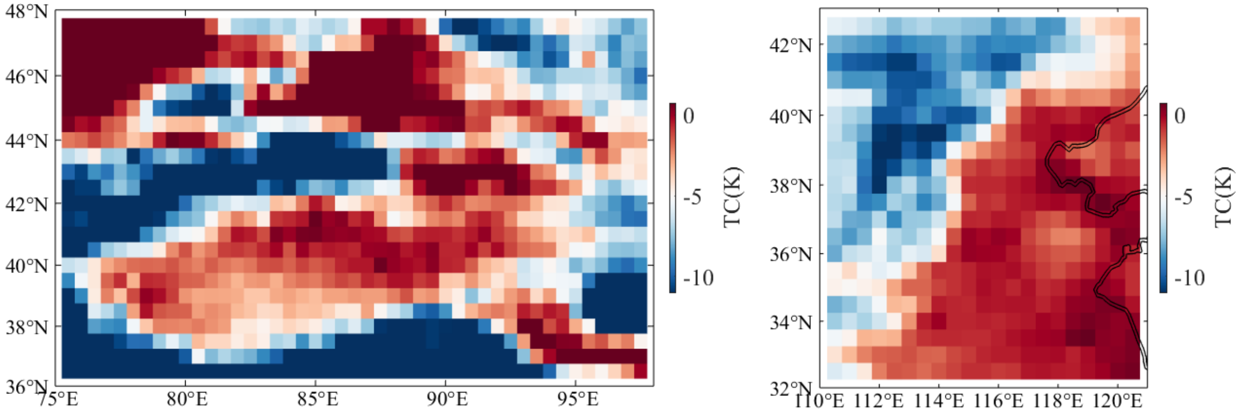

Figure S7. Spatial distribution of monthly mean TCs in western China and the North China Plain.

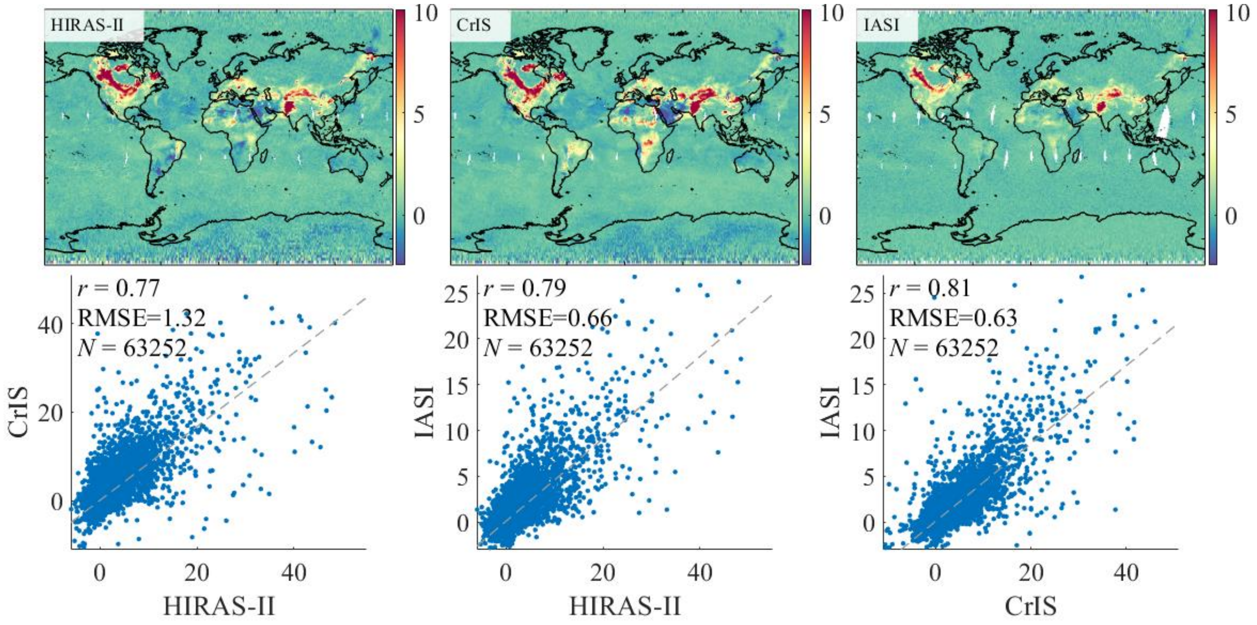

Figure S8. Top panel: NH_3 HRI derived from FY-3E/HIRAS-II, SNPP/CrIS, and Metop-B/IASI on July 15, 2023, respectively. HIRAS-II and CrIS have the same spectral resolution (0.625 cm^{-1}) and coincident spectral channels, so we use the same background measurements (as described in Text S1) to calculate the HRI from both sounders. The spectral resolution of IASI (0.25 cm^{-1}) is inconsistent with HIRAS-II and CrIS, but fortunately, the official IASI product provides the HRI for NH_3, so we use that directly. Therefore, the difference between the HRI of HIRAS-II and CrIS comes only from the instruments themselves (radiometric performance and overpass time), and the difference with IASI also includes the different background measurements taken by IASI. Bottom: The relationship between the HRIs of the three sounders. The HRI here results from averaging the unit latitude and longitude grids.

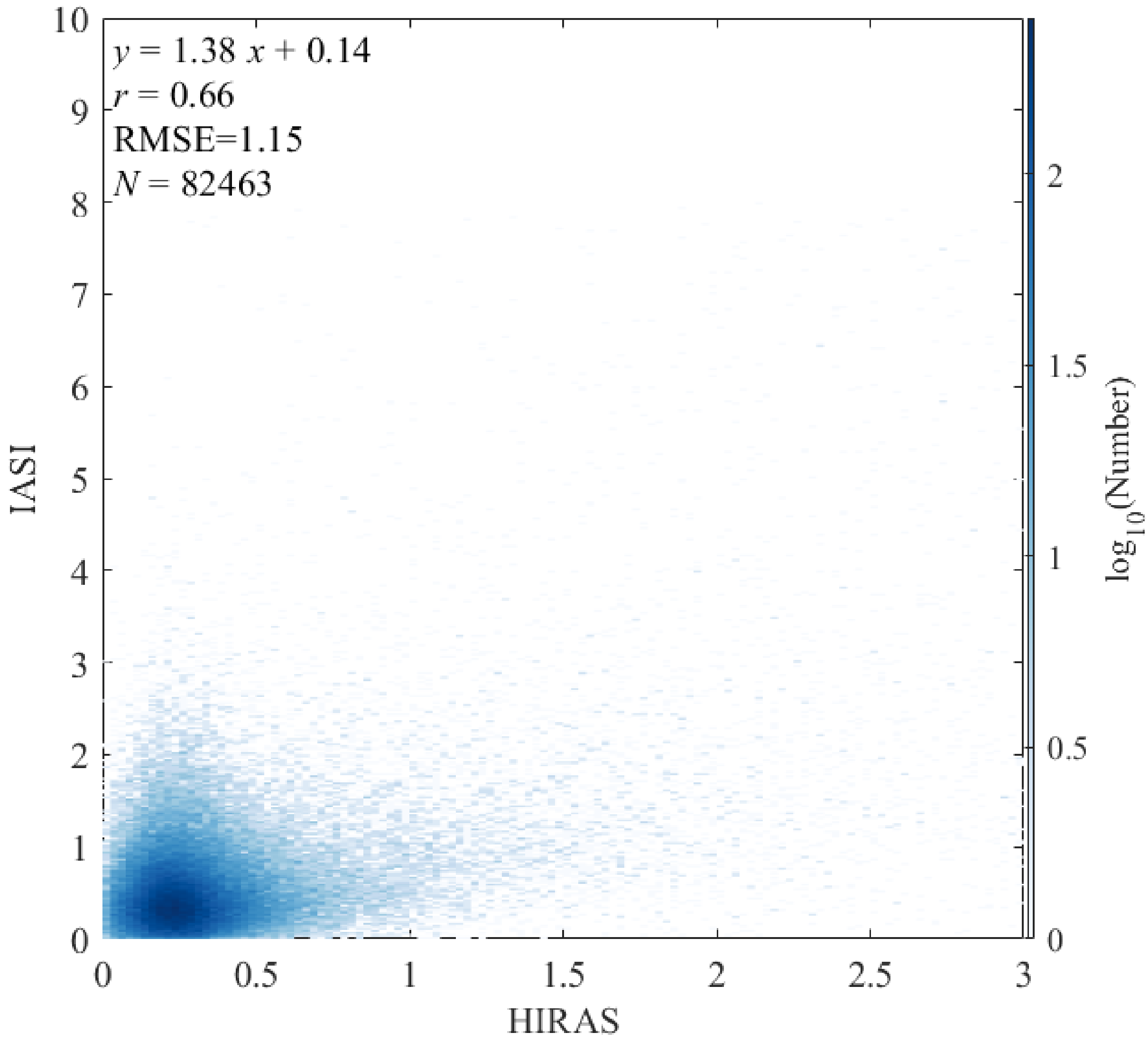

Figure S9. Relationship between HIRAS-II and IASI NH_3 grid-averaged global columns on July 15, 2023.

References

(1) Carn, S. A.; Strow, L. L.; de Souza-Machado, S.; Edmonds, Y.; Hannon, S. Quantifying Tropospheric Volcanic Emissions with AIRS: The 2002 Eruption of Mt. Etna (Italy). *Geophys. Res. Lett.* **2005**, *32* (2). https://doi.org/10.1029/2004GL021034.

(2) Beer, R.; Shephard, M. W.; Kulawik, S. S.; Clough, S. A.; Eldering, A.; Bowman, K. W.; Sander, S. P.; Fisher, B. M.; Payne, V. H.; Luo, M.; Osterman, G. B.; Worden, J. R. First Satellite Observations of Lower Tropospheric Ammonia and Methanol. *Geophys. Res. Lett.* **2008**, *35* (9). https://doi.org/10.1029/2008GL033642.

(3) Coheur, P.-F.; Clarisse, L.; Turquety, S.; Hurtmans, D.; Clerbaux, C. IASI Measurements of Reactive Trace Species in Biomass Burning Plumes. *Atmos. Chem. Phys.* **2009**, *9* (15), 5655–5667.
(4) Clarisse, L.; R'Honi, Y.; Coheur, P.-F.; Hurtmans, D.; Clerbaux, C. Thermal Infrared Nadir Observations of 24 Atmospheric Gases. *Geophys. Res. Lett.* **2011**, *38* (10). https://doi.org/10.1029/2011GL047271.
(5) Clarisse, L.; Coheur, P.-F.; Chefdeville, S.; Lacour, J.-L.; Hurtmans, D.; Clerbaux, C. Infrared Satellite Observations of Hydrogen Sulfide in the Volcanic Plume of the August 2008 Kasatochi Eruption. *Geophys. Res. Lett.* **2011**, *38* (10). https://doi.org/10.1029/2011GL047402.
(6) Fu, D.; Millet, D. B.; Wells, K. C.; Payne, V. H.; Yu, S.; Guenther, A.; Eldering, A. Direct Retrieval of Isoprene from Satellite-Based Infrared Measurements. *Nat. Commun.* **2019**, *10* (1), 3811. https://doi.org/10.1038/s41467-019-11835-0.
(7) De Longueville, H.; Clarisse, L.; Whitburn, S.; Franco, B.; Bauduin, S.; Clerbaux, C.; Camy-Peyret, C.; Coheur, P.-F. Identification of Short and Long-Lived Atmospheric Trace Gases From IASI Space Observations. *Geophys. Res. Lett.* **2021**, *48* (5), e2020GL091742. https://doi.org/10.1029/2020GL091742.
(8) An, N.; Shang, H.; Lesi, W.; Ri, X.; Shi, C.; Tana, G.; Bao, Y.; Zheng, Z.; Xu, N.; Chen, L.; Zhang, P.; Ye, L.; Letu, H. A Cloud Detection Algorithm for Early Morning Observations From the FY-3E Satellite. *IEEE Trans. Geosci. Remote Sens.* **2023**, *61*, 1–15. https://doi.org/10.1109/TGRS.2023.3304985.
(9) Rodgers, C. D. *Inverse Methods for Atmospheric Sounding*; WORLD SCIENTIFIC, 2000. https://doi.org/10.1142/3171.
(10) Whitburn, S.; Van Damme, M.; Clarisse, L.; Bauduin, S.; Heald, C. L.; Hadji-Lazaro, J.; Hurtmans, D.; Zondlo, M. A.; Clerbaux, C.; Coheur, P.-F. A Flexible and Robust Neural Network IASI-NH3 Retrieval Algorithm. *J. Geophys. Res.: Atmos.* **2016**, *121* (11), 6581–6599. https://doi.org/10.1002/2016JD024828.
(11) Clarisse, L.; Franco, B.; Van Damme, M.; Di Gioacchino, T.; Hadji-Lazaro, J.; Whitburn, S.; Noppen, L.; Hurtmans, D.; Clerbaux, C.; Coheur, P. The IASI NH_3 Version 4 Product: Averaging Kernels and Improved Consistency. *Atmos. Meas. Tech. Discuss.* **2023**, *2023*, 1–31. https://doi.org/10.5194/amt-2023-48.
(12) Van Damme, M.; Clarisse, L.; Franco, B.; Sutton, M. A.; Erisman, J. W.; Kruit, R. W.; Van Zanten, M.; Whitburn, S.; Hadji-Lazaro, J.; Hurtmans, D. Global, Regional and National Trends of Atmospheric Ammonia Derived from a Decadal (2008–2018) Satellite Record. *Environ. Res. Lett.* **2021**, *16* (5), 055017.
(13) Van Damme, M.; Whitburn, S.; Clarisse, L.; Clerbaux, C.; Hurtmans, D.; Coheur, P.-F. Version 2 of the IASI NH3 Neural Network Retrieval Algorithm: Near-Real-Time and Reanalysed Datasets. *Atmos. Meas. Tech.* **2017**, *10* (12), 4905–4914. https://doi.org/10.5194/amt-10-4905-2017.
(14) Brewer, J. F.; Millet, D. B.; Wells, K. C.; Payne, V. H.; Kulawik, S.; Vigouroux, C.; Cady-Pereira, K. E.; Pernak, R.; Zhou, M. Space-Based Observations of Tropospheric Ethane Map Emissions from Fossil Fuel Extraction. *Nat. Commun.* **2024**, *15* (1), 7829. https://doi.org/10.1038/s41467-024-52247-z.
(15) Franco, B.; Clarisse, L.; Theys, N.; Hadji-Lazaro, J.; Clerbaux, C.; Coheur, P. Pyrogenic HONO Seen from Space: Insights from Global IASI Observations. *Atmos. Chem. Phys.* **2024**, *24* (8), 4973–5007.

(16) Franco, B.; Clarisse, L.; Van Damme, M.; Hadji-Lazaro, J.; Clerbaux, C.; Coheur, P.-F. Ethylene Industrial Emitters Seen from Space. *Nat. Commun.* **2022**, *13* (1), 6452. https://doi.org/10.1038/s41467-022-34098-8.

(17) Franco, B.; Clarisse, L.; Stavrakou, T.; Müller, J.-F.; Taraborrelli, D.; Hadji-Lazaro, J.; Hannigan, J. W.; Hase, F.; Hurtmans, D.; Jones, N.; Lutsch, E.; Mahieu, E.; Ortega, I.; Schneider, M.; Strong, K.; Vigouroux, C.; Clerbaux, C.; Coheur, P.-F. Spaceborne Measurements of Formic and Acetic Acids: A Global View of the Regional Sources. *Geophys. Res. Lett.* **2020**, *47* (4), e2019GL086239. https://doi.org/10.1029/2019GL086239.

(18) Franco, B.; Clarisse, L.; Stavrakou, T.; Müller, J.-F.; Pozzer, A.; Hadji-Lazaro, J.; Hurtmans, D.; Clerbaux, C.; Coheur, P.-F. Acetone Atmospheric Distribution Retrieved From Space. *Geophys. Res. Lett.* **2019**, *46* (5), 2884–2893. https://doi.org/10.1029/2019GL082052.

(19) Franco, B.; Clarisse, L.; Stavrakou, T.; Müller, J.-F.; Van Damme, M.; Whitburn, S.; Hadji-Lazaro, J.; Hurtmans, D.; Taraborrelli, D.; Clerbaux, C. A General Framework for Global Retrievals of Trace Gases from IASI: Application to Methanol, Formic Acid, and PAN. *J. Geophys. Res.: Atmos.* **2018**, *123* (24), 13,963-13,984.

(20) Rosanka, S.; Franco, B.; Clarisse, L.; Coheur, P.-F.; Pozzer, A.; Wahner, A.; Taraborrelli, D. The Impact of Organic Pollutants from Indonesian Peatland Fires on the Tropospheric and Lower Stratospheric Composition. *Atmos. Chem. Phys.* **2021**, *21* (14), 11257–11288. https://doi.org/10.5194/acp-21-11257-2021.

(21) Wells, K.; Millet, D.; Brewer, J.; Payne, V.; Cady-Pereira, K.; Pernak, R.; Kulawik, S.; Vigouroux, C.; Jones, N.; Mahieu, E.; Makarova, M.; Nagahama, T.; Ortega, I.; Palm, M.; Strong, K.; Schneider, M.; Smale, D.; Sussmann, R.; Zhou, M. Long-Term Global Measurements of Methanol, Ethene, Ethyne, and HCN from the Cross-Track Infrared Sounder. *EGUsphere* **2024**, *2024*, 1–40. https://doi.org/10.5194/egusphere-2024-1551.

(22) Wells, K. C.; Millet, D. B.; Payne, V. H.; Vigouroux, C.; Aquino, C. A. B.; De Mazière, M.; de Gouw, J. A.; Graus, M.; Kurosu, T.; Warneke, C.; Wisthaler, A. Next-Generation Isoprene Measurements From Space: Detecting Daily Variability at High Resolution. *J. Geophys. Res.: Atmos.* **2022**, *127* (5), e2021JD036181. https://doi.org/10.1029/2021JD036181.
